# Supplementary material for: High-Quality Amorphous Silicon Carbide for Hybrid Photonic Integration Deposited at a Low Temperature
Source: ACS Photonics. 2023 Sep 21;10(10):3748–54. doi: 10.1021/acsphotonics.3c00968 (PMC10588551; doi:10.1021/acsphotonics.3c00968)
Supplement: Supplementary file 1 — ph3c00968_si_001.pdf [file ph3c00968_si_001.pdf]

HIGH QUALITY AMORPHOUS SILICON CARBIDE FOR HYBRID PHOTONIC INTEGRATION DEPOSITED AT LOW TEMPERATURE: SUPPLEMENTARY INFORMATION

Bruno Lopez-Rodriguez,∗,†,‖ Roald van der Kolk,‡,‖ Samarth Aggarwal,¶,‖ Naresh Sharma,† Zizheng Li,† Daniel van der Plaats,† Thomas Scholte,† Jin Chang,§ Simon Gr¨oblacher,§ Silvania F. Pereira,† Harish Bhaskaran,¶ and Iman Esmaeil Zadeh†

†Department of Imaging Physics (ImPhys), Faculty of Applied Sciences, Delft University of Technology, Delft 2628 CJ, The Netherlands
‡Kavli Institute of Nanoscience, Delft University of Technology, Delft 2628 CD, The Netherlands
¶Department of Materials, University of Oxford, Parks Road, Oxford OX1 3PH, U.K.
§Department of Quantum Nanoscience, Faculty of Applied Sciences, Delft University of Technology, Delft 2628 CJ, The Netherlands
‖The authors contributed equally to this work

Deposition recipes and ellipsometry data

The deposition of amorphous silicon carbide films in PECVD was done using a mixture of SiH_4 (15 sccm), CH_4 (75 sccm) and Ar (285 sccm) with temperatures ranging between 300°C and 400°C. The chamber pressure was kept in all recipes at 1000 mTorr with a forward plasma power (P_{FW}) of 20W. The average deposition rates were 39 nm/min. An example for the effect in optical properties (refractive index and extinction coefficient) upon the variation of Si/C ratio during PECVD deposition is shown in fig.S1.

For the deposition of ICPCVD a-SiC films, the same mixture was applied with 15 sccm of SiH_4, 10 sccm of CH_4, at temperatures ranging between 150°C and 400°C. In this case, an Ar flow close to the sample (Ar_{ring}) was choose to be 11 sccm while the Ar flow in the camber was 10 sccm. The chamber pressure was kept at 2 mTorr for all recipes with a forward plasma power of 750 W. The average deposition rates were 37 nm/min. In fig.S2 it can be seen that lowering the temperature lowers the roughness in the films with a minimum at 150°C.

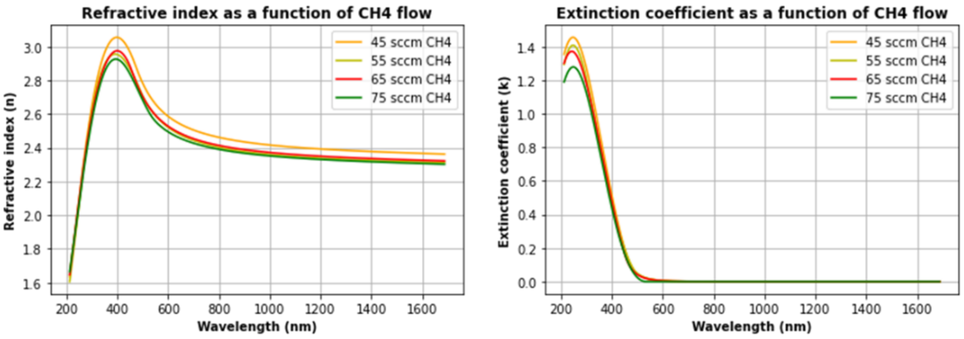

Fig. S1. Refractive index and loss coefficient of a-SiC films deposited at 300°C with PECVD as a function of methane flow for a fixed silane flow.

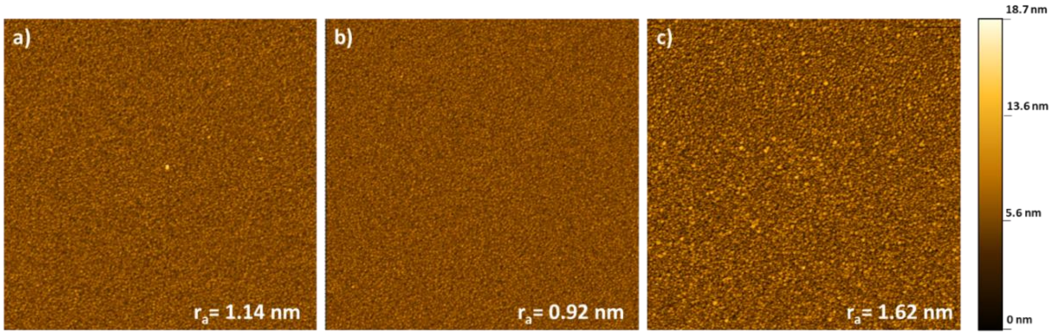

Fig. S2. Atomic Force microscope images of ICPCVD a-SiC deposited at a) 75°C, b) 150°C and c) 400°C

Device fabrication and inspection

The development of the electron beam resist was performed in a three-step process immersing the samples in pentyl-acetate, MBIK:IPA (1:1) and IPA for 1 min each. For etching, the plasma power was set to 20W with 13.5 sccm of SF_6 and 3.5 sccm of O_2 at a chamber pressure of 8 μBar. After etching the excess e-beam resist was removed by oxygen plasma cleaning (200 sccm O_2 at 50W) for 8 minutes.

To determine the specific dimensions of the waveguides under study, electron microscope images were taken top view and cross-section as shown in fig.S3.

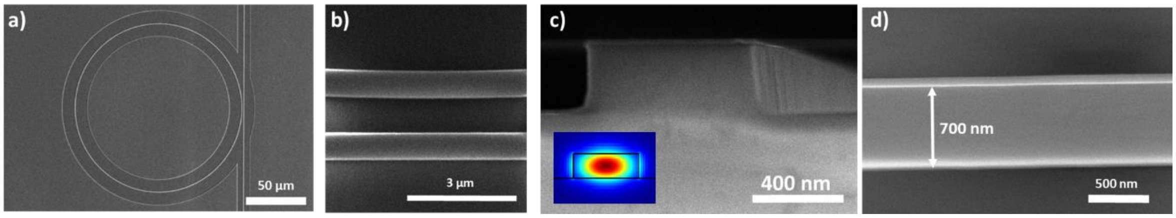

Fig. S3. Scanning Electron Microscope images of a) a ring resonator with 100 μm radius and gap 900 nm, b) gap region between the ring resonator and the bus waveguide, c) side image of the waveguide profile and d) top image of the bus waveguide for a design of 800 nm width.

Determination of quality factor and waveguide propagation losses

The most important parameter in an optical ring resonator is the loaded quality factor (Q_L), and is obtained experimentally from the transmission spectra of a resonance dip (or peak). This value quantifies the filtering quality of the device, and it is defined as [S1]:

$$Q_L = \frac{\lambda}{\Delta\lambda} \qquad (S1)$$

where λ is the center wavelength at the resonance and $\Delta\lambda$ is the Full Width at half maximum of the Lorentzian fit. However, since this value is affected by the interaction with the coupling waveguide, the following expression for an intrinsic quality factor is often preferred:

$$Q_i = \frac{2Q_L}{1+\sqrt{T}} \qquad (S2)$$

In this equation, T denotes the transmission at the resonance.

From the intrinsic quality factor (Q_i), the loss coefficient (α) can be estimated by attending to the following expressions [S2]:

$$\alpha = \frac{2\pi n_g}{Q_i \lambda} \qquad (S3)$$

where the group index (n_g) can be determined using experimental data of the free spectral range (FSR) of the ring resonator with radius R under study:

$$n_g = \frac{\lambda^2}{2\pi R \cdot FSR} \qquad (S4)$$

Last, to convert to units of dB/cm, the relation below is applied to the loss coefficient (cm^{-1}) , with L being the length of the waveguide:

$$\alpha\left(\frac{dB}{cm}\right) = \frac{1}{L} 10log_{10}\left(\frac{P}{P_o}\right) = \frac{1}{L} 10log_{10}(e^{-\alpha L}) = 4.3429 \cdot \alpha(cm^{-1}) \qquad (S5)$$

Systematic measurements of quality factor, free spectral range and group index

To systematically characterize the devices with PMMA cladding, we used a C-L band tunable laser (Santec TSL-550). The light is coupled into the device using grating couplers patterned in the films. A fiber polarization controller Thorlabs (FPC032) is used to select the polarization of the input light. To obtain the transmission spectrum of the optical ring resonators, the wavelength of the laser is swept from 1500-1600 nm with 1 pm resolution at a speed of 10 nm/sec. The output transmission of the device is collected on a photodetector (Newport 2011). The following data is taken for the best two samples in the several deposition runs of ICPCVD. For these systematic measurements, devices were fabricated including grating couplers and an automatic setup was used to obtain the spectra. For reference, PECVD is also shown to demonstrate and compare this technique. In the later, the temperature cannot be lower than 200°C since no functional devices will be achieved. All the results obtained for the best two recipes in ICPCVD and PECVD are shown in fig.S4. Devices patterned on PECVD films deposited at 400°C presented higher quality factors, thus confirming that the main loss mechanism in PECVD, hydrogen incorporation, is lower. In this case, the highest loaded quality factor for the ring resonator was 1.8x10^5 with intrinsic quality factor of 2.1x10^5. On the other hand, ICPCVD shows a great stability over a broader temperature range due to the minimal incorporation of hydrogen and generally, lower grain sizes. The device with the highest quality factor obtained during this optimization process is shown in fig.S5.

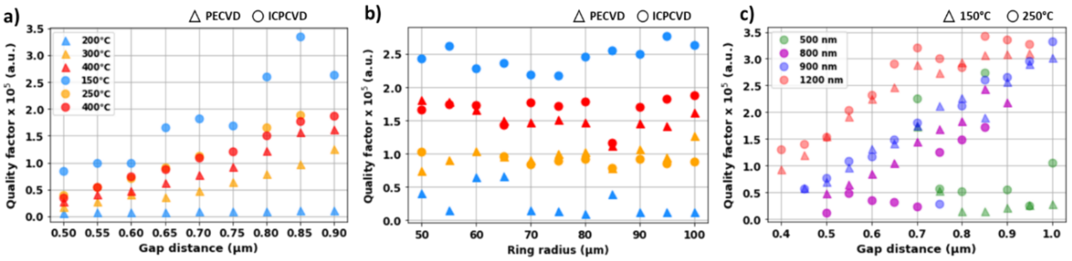

Fig. S4. Systematic data acquisition for the loaded quality factor of ring resonators in PECVD (400°C) and ICPCVD (150°C) as a function of a) gap distance and b) ring radius; c) gap distance for different waveguides widths and two different temperatures of ICPCVD.

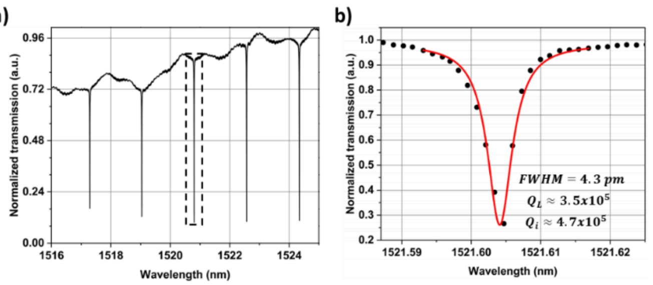

Fig. S5. a) Transmission spectrum from 1516 to 1525 nm of the ring resonator with highest quality factor with a free spectral range of 1.4 and b) 1 pm resolution scan of a resonance dip at 1521.65 nm.

For the device shown in fig.S5 and using the equations introduced in the beginning, a waveguide propagation loss of 1.06 dB/cm is obtained. Nevertheless, the lowest propagation losses were obtained for the device with 150 μm radius and PMMA cladding shown in fig. S6 with 0.78 dB/cm.

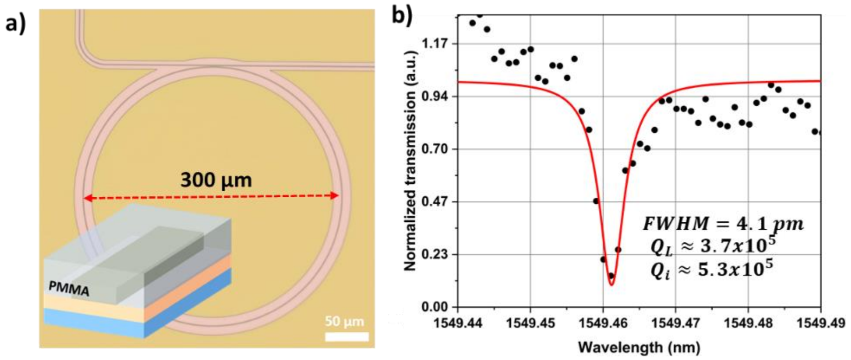

Fig. S6. a) Optical microscope image of a ring resonator with 300 μm diameter fabricated on a-SiC films deposited at 150°C with PMMA cladding and b) normalized transmission spectra with 1 pm resolution of a resonance dip around 1549.46 nm.

The free spectral range of the devices was taken from a set of dips around 1550 nm and the measurement number indicates each of the dip to dip distances. The group index was obtained using eq.S4 for each wavelength and ring radius and the data is represented in fig.S7.

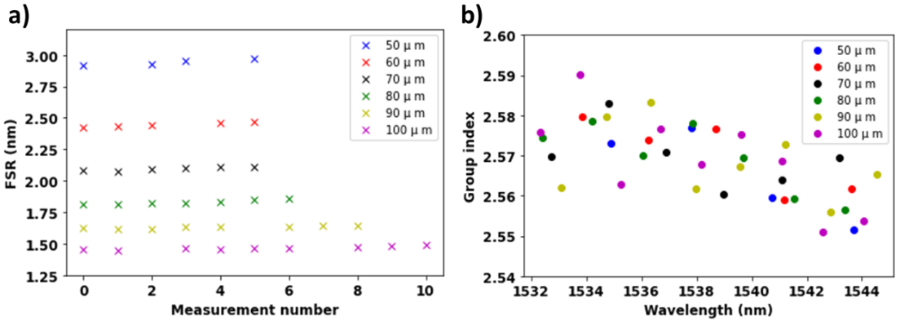

Fig. S7. For devices made on ICPCVD a-SiC deposited at 150°C with different radii, from 50 µm (blue) to 100 µm (purple), a) Free spectral range of several peaks around 1550 nm and b) group index as a function of the wavelength obtained from the FSR data for different radius.

Thermo-optic coefficient of a-SiC devices

To determine the thermo-optic coefficient (TOC) of the optical ring resonators, the refractive index (n) and the thickness of the film (t) was obtained from ellipsometry data (shown in fig.S8) and confirmed via electron microscope images of the devices under study. Finite-Domain Time-Domain (FDTD) simulations using the commercial software Lumerical were performed to obtain the effective refractive index (n_{eff}) and the overlapping factor (Γ).

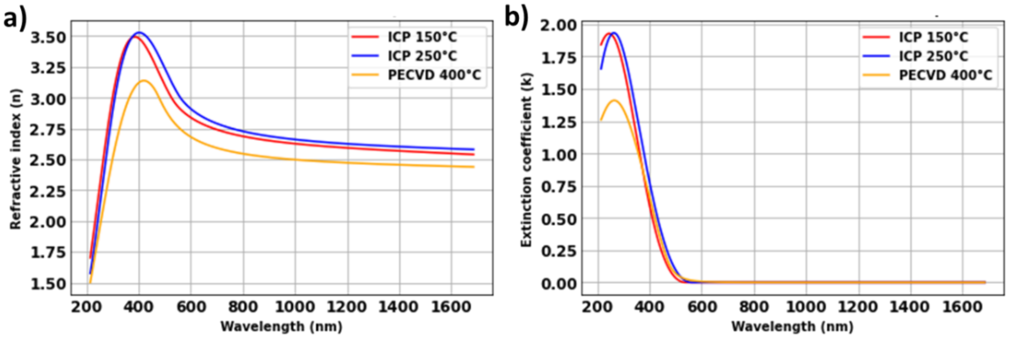

Fig. S8. Ellipsometry data for ICPCVD films deposited at 150°C and 250°C and PECVD deposited at 400°C showing a) refractive index and b) extinction coefficient

The samples were placed on top of a PCB using thermally conductive silver paste (fig.S9a). The PCB has a resistive thermal element and the temperature can be varied with a voltage supply up to 100°C (fig.S9b). The temperature is recorded using a temperature sensor close to the sample surface. Optical access to the devices is performed with the same polarization maintaining fiber v-groove assemblies used for the initial characterization. In table S1 are shown the main parameters used for the calculation of the TOC together with the loaded and intrinsic quality factors for the best performing recipes.

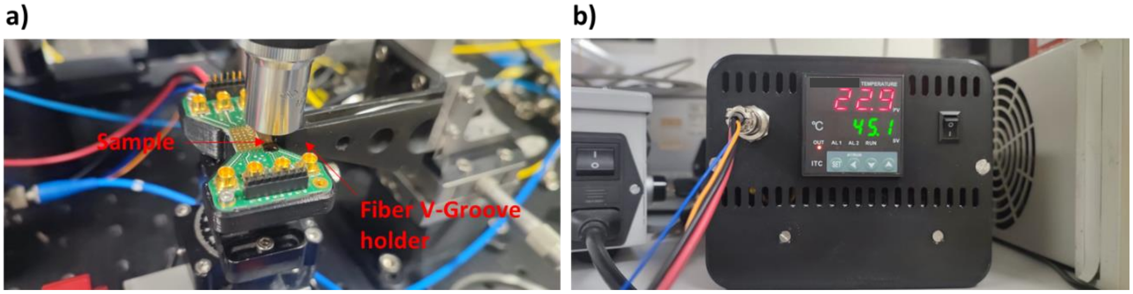

Fig. S9. a) PCB with heating element and b) temperature controller for side coupling configuration.

Sample	t (nm)	n_{eff}	Shift (pm/°C)	TOC (10^{-5}/°C)	Q loaded ($x10^5$)	Q intrinsic ($x10^5$)

ICP 150°C	280	1.91	36.2	7.3	4.2	5.7
ICP 250°C	270	1.92	36.6	7.4	1.9	2.4
PECVD 400°C	265	1.79	28.8	5.1	1.8	2.1

Table S1. Physical and optical parameters of fabricated devices.

Lift-off of a-SiC devices

The sample is prepared by exposing a 1 μm layer of PMMA with the desired shape to transfer. The shape used in this study is a squared island, yet the feasibility of this method is not limited to the geometry to transfer, enabling for small footprint and high flexibility for the implementation. Afterwards, deposition of a-SiC at 150°C is performed, and the pattern is revealed by doing lift-off with acetone at 53°C, followed by a patterning step for the optical devices that can be seen in fig.S10a and are tested with a side coupling configuration.

A close look of the interface between the deposited a-SiC and the thermal SiO_2 in fig.10c shows that the temperature at which the films are deposited does not affect the quality of the lift-off. Devices fabricated on films deposited without the lift-off technique showed similar performance and the degradation can be attributed to the deposition tool as shown in fig.10b.

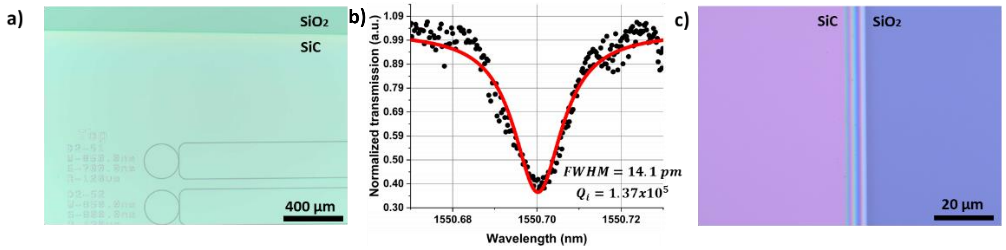

Fig. S10. a) Optical microscope image of lift-off a-SiC deposited at 150°C with ring resonator devices, b) 1pm resolution transmission spectra of one of the devices and c) edge of the region between a-SiC and the substrate.

Taper design for a-SiC-SiN integration

To determine the losses between the two platforms we performed Lumerical simulations considering the overlapping integral of the power in the two waveguides (fig.S11a for a-SiC profile and fig.S11b for 40 nm SiN). For the confinement of the final mode in the a-SiC, several bending radii were chosen and the power loss after the bend was evaluated and shown fig.S12a with the configuration in fig.S12b. The monitor size to compute this power transfer was set to 1.2x1μm^2 .

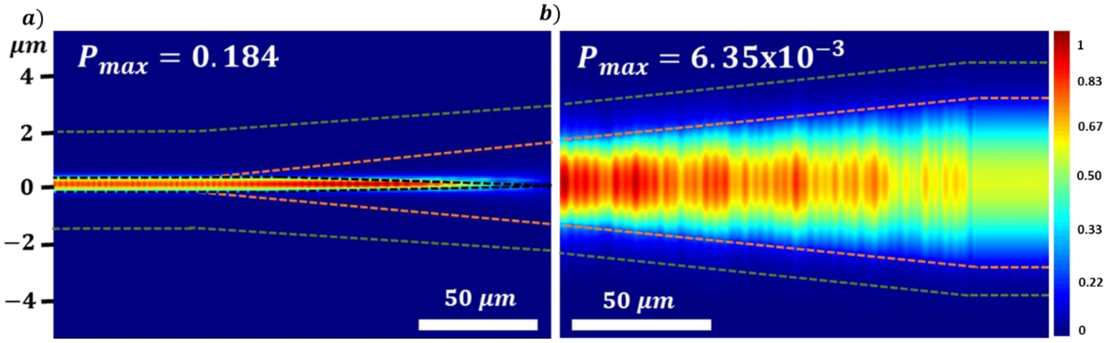

Fig. S11. Power distribution obtained by FDTD Simulations of the top taper region of a) a-SiC (dotted black line) and b) SiN (dotted orange line): both embedded in a tapered SiO_2 cladding (dotted green line).

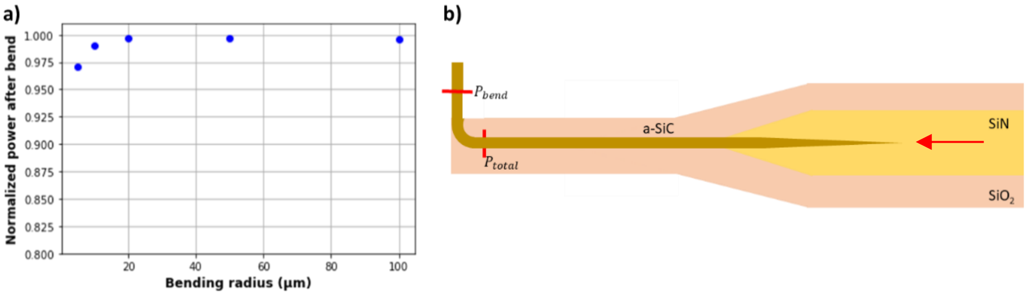

Fig. S12. a) Normalized power transfer as a function of bending radius and b) taper and monitor position for the determination of the bending losses.

Fabrication route for integration of a-SiC and lithium niobate

To protect the lithium niobate layer to the etching chemistry of a-SiC, a thin layer of silicon dioxide (50 nm) is deposited via PECVD or ICPCVD. In case the roughness of this layer affects the optical performance of the a-SiC devices, a planarization step using ion beam etching can be followed. Deposition of a-SiC films at low temperature is performed with a thickness of 280 nm. Depending on the configuration needed for modulation, a second etching step can be performed using Ar milling to define a waveguide in the LN or to etch a bias for the Au contact pads, which could enhance the modulation. After etching the LN waveguide, RCA-1 cleaning step is used to remove organic contaminants and reduce the sidewall roughness of the optical devices, enhancing the optical performance. For the FDTD simulations, the refractive index of a-SiC was chosen to be n=2.589 according to experimental data obtained via ellipsometry while for x-cut Lithium Niobate, it was chosen as n_x=2.21, n_y=2.21 and n_z=2.13 (according to the coordinates in fig.S13b).

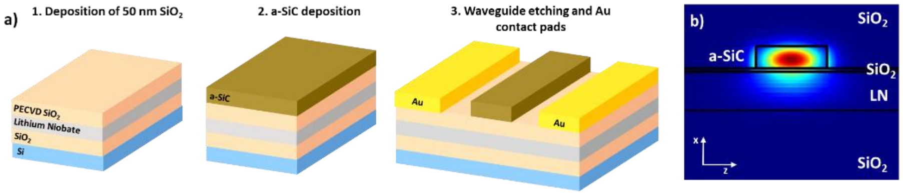

Fig. S13. a) Fabrication flow of a-SiC on LN for electro-optic modulation and b) mode profile obtained via FDTD 3D simulations of a-SiC/LN. The isolation layer between a-SiC and LN allows for the etching of the a-SiC waveguides without affecting the performance of the LN.

Comparison of different optical platforms

Material	Refractive index (n)	Width / thickness (nm)	Qint x 10^5 / losses (dB/cm)	Temperature (°C)	Kerr coefficient n_2 (cm^2/W)	Reference
PECVD SiN	1.9	1500/730	7.24 / 0.42	350	-	[S3]
LPCVD SiN	2.07	1300 / 655	8 / 0.4	700-850/1100*	$5\cdot 10^{-15}$	[S4]
Sputtered AlN	2.02	3500/1100	37 / 0.1	300	-	[S5]
3C-SiCOI	2.6	1700/500	1.42 / 2.9	-	-	[S6]
4H-SiCOI	2.6	3000/530	11 / 0.38	-	$6.9 \cdot 10^{-15}$	[S7]
4H-SiCOI	2.6	1850/500-600	56 / -	-	$6.9 \cdot 10^{-15}$	[S8]
PECVD a-SiC	2.45	800/350	1.6 / 3	300	4.8×10^{-14}	[S9]
**ICPCVD a-SiC**	**2.589**	**750/280**	**4.7-5.7 / 0.78-1.07**	**150**	**-**	**This work**

Table S2. Physical and optical parameters of fabricated devices. *Annealing temperature

References

[S1] P. E. Barclay, K. Srinivasan, and O. Painter, “Nonlinear response of silicon photonic crystal microresonators excited via an integrated waveguide and fiber taper,” Opt. Express 13, 801–820 (2005).

[S2] P. Rabiei, W. H. Steier, C. Zhang, and L. R. Dalton, “Polymer micro-ring filters and modulators,” J. Light. Technol 20, 1968 (2002).

[S3] Ji, X.; Okawachi, Y.; Gil-Molina, A.; Corato-Zanarella, M.; Roberts, S.; Gaeta, A. L.;Lipson, M. Ultra-Low-Loss Silicon Nitride Photonics Based on Deposited Films Compatible with Foundries. Laser & Photonics Reviews 2023, 17, 2200544

[S4] Ye, Z.; F¨ul¨op, A.; ´Oskar Bjarki Helgason,; Andrekson, P. A.; Torres-Company, V. Low-loss high-Q silicon-rich silicon nitride microresonators for Kerr nonlinear optics. Opt.Lett. 2019, 44, 3326–3329

[S5] Liu K, Yao S, Ding Y, Wang Z, Guo Y, Yan J, Wang J, Yang C, Bao C. Fundamental linewidth of an AlN microcavity Raman laser. Opt Lett. 2022 Sep 1;47(17):4295-4298

[S6] Tianren Fan, Hesam Moradinejad, Xi Wu, Ali A. Eftekhar, and Ali Adibi, "High-Q integrated photonic microresonators on 3C-SiC-on-insulator (SiCOI) platform," Opt. Express 26, 25814-25826 (2018)

[S7] Guidry, M. A.; Yang, K. Y.; Lukin, D. M.; Markosyan, A.; Yang, J.; Fejer, M. M.;Vuckovic, J. Optical Parametric oscillation in silicon carbide nanophotonics. Optica 2020, 7, 1139–1142.

[S8] Guidry, M. A.; Lukin, D. M.; Yang, K. Y.; Trivedi, R.; Vuckovic, J. Quantum optics of soliton microcombs. Nature Photonics 2021, 16, 52–58

[S9] Xing, P.; Ma, D.; Ooi, K. J. A.; Choi, J. W.; Agarwal, A. M.; Tan, D. CMOS-Compatible PECVD Silicon Carbide Platform for Linear and Nonlinear Optics. ACS Photonics 2019, 6, 1162–1167.
